# The Effect of Patellar Taping on Squat Depth and the Perception of Pain in People with Anterior Knee Pain

**DOI:** 10.2478/hukin-2013-0031

**Published:** 2013-07-05

**Authors:** Amanda M. Clifford, Elaine Harrington

**Affiliations:** 1 Department of Clinical Therapies,Faculty of Education and Health Sciences, University of Limerick, Ireland.

**Keywords:** anterior knee pain, patellar taping, squat

## Abstract

Patellar taping is a treatment adjunct commonly used in the management of anterior knee pain. The aim of this cross sectional study was to investigate the effects of medial glide patellar taping on sagittal plane lower-limb joint kinematics and knee pain during a unilateral squat in a symptomatic population complaining of anterior knee pain. Ten participants with a history of unilateral or bilateral anterior knee pain were included in the study. Subjects were required to squat on the symptomatic leg under three conditions: placebo tape, patellar tape and no tape. Kinematic data was recorded using the CODA mpx64 motion analysis system and subjects’ pain was assessed using the Numerical Rating Scale. Patellar taping resulted in a significantly greater single-legged squat depth compared to placebo tape (p=0.008) and no tape (p=0.001) and a statistically significant reduction in pain during a squat compared to placebo tape (p=0.001) or no tape (p=0.001). Significant differences were not identified for maximum knee flexion in the patella taping compared to the no tape condition. This study may have significant clinical implications as participants reported less pain and alterations in sagittal plane movement following the application of patellar tape.

## Introduction

Anterior knee pain (AKP) is a common symptom complex typically characterized by diffuse retropatellar or peripatellar knee pain exacerbated by activities that load the flexed knee joint. Such activities include ascending or descending stairs, squatting, walking, running or sitting for prolonged periods of time ( Fulkerson, 2002 ). The literature suggests that patients with longstanding AKP have an associated lateral displacement of the patella within the femoral trochlea groove ( Merchant, 1988 ; Crossley et al., 2000 ; Herrington and Nester, 2004 ). This may be a result of impaired activation or timing of vastus medialis oblique (VMO) in relation to vastus lateralis (VL), which contributes to increased stresses at the patellofemoral joint ( Crossley et al., 2000 ). Authors have reported kinematic alterations in individuals with anterior knee pain including reduced knee flexion during functional weight-bearing activities. These compensatory movement patterns are thought to be adopted by people with AKP in order to reduce the load across the patellofemoral joint, reducing subsequent demand on the quadriceps ( Greenwald et al., 1996 ; Nadeau et al., 1997 ). Reduced activation of the quadriceps in this manner leads to weakness of VMO and VL, a common clinical finding in patients with AKP ( Thomee et al., 1999 ). This in turn may lead to altered stabilisation of the patella and further patellofemoral joint dysfunction ( Crossley et al., 2000 ).

Most patients with AKP respond favourably to conservative management such as exercise ( Boling et al., 2006 ; Philips and Coetsee, 2008 ) and patella taping ( Warden et al., 2007 ). Although the efficacy of an exercise-based rehabilitation programme as the primary treatment for AKP for improvements in pain and function has been widely established ( Clark et al., 2000 ; Crossley et al., 2002 ; Whittingham et al., 2004 ; Boling et al., 2006 ; Philips and Coetsee, 2008 ), evidence to support the advocacy of patella taping is inconclusive. The proposed mechanisms by which patellofemoral joint taping works is by facilitating the activation of VMO (the main active stabiliser of the patella), reducing the typically predominant lateral pull of VL ( McConnell, 1986 ), and repositioning the patella within the patellofemoral trochlear groove, altering patella tracking through a change in the quadriceps lever arm ( Herrington and Nester, 2004 ).

To date, the current literature regarding alterations in lower-limb kinematics following taping is inconsistent. Positive effects have been found in some parameters including an increase in cadence, and knee flexion angles during stair ascent and descent ( Salsich et al., 2001 ). However, significant changes were not reported in stride characteristics (except for stride length) or in a vertical jumping task ( Aminaka and Gribble, 2005 ). No known published study has examined the effect of patella taping on lower-limb kinematics during a squat. The single-leg squat is regularly selected clinically as an objective measure for its potential to detect deficits in muscle strength and motor control ( Zeller et al., 2003 ). It is also reported to simulate athletic postures frequently assumed during several sporting activities ( Zeller et al., 2003 ) and is commonly reported as a painful activity in people with AKP ( Fulkerson, 2002 ). Also, the single legged squat is an advanced exercise used in many rehabilitation programmes for AKP, as it is one of the most beneficial strengthening exercises for both concentric and eccentric control at the knee joint ( Donnelly et al., 2006 ). Thus, it is hypothesised that the assessment of pain and sagittal plane kinematics during the single-legged squat both with and without patella tape may be useful outcome measures to predict return to sport or return to baseline level of function. The objective of this study was to investigate the effects of patella taping on sagittal plane kinematics during a single-legged squat. A second objective was to investigate any subsequent change in pain during a squat with and without tape. The null hypothesis of this study was that taping would not have any effect on kinematics or pain during a single-leg squat.

## Material and Methods

### Participants

Ethical approval was obtained for the study from the University of Limerick’s Research Ethics Committee and written consent was obtained from each participant prior to data collection. In accordance with the literature ( Thomee et al., 1999 ), subjects that were currently complaining of AKP of gradual onset and lasting six months or longer were recruited to participate in the study. AKP was defined as a pain or ache at the front of the knee that occurs during one or more of the following activities; sitting for long periods of time such as in the cinema or while driving, ascending or descending stairs, running, squatting, jumping or kneeling ( Fulkerson, 2002 ). Volunteers were excluded from the study if they were under eighteen years of age, had any history of subluxation or dislocation of the patella, anterior or posterior ligament insufficiency, Osgood Schlatter’s disease, previous knee surgery or meniscal damage; in order to rule out similar pathologies and confounding variables in line with previous research ( Clark et al., 2000 ; Crossley et al., 2002 ; Whittingham et al., 2004 ). Volunteers with any other underlying musculoskeletal problems that would have prevented the subject from completing the study were also excluded. In order to achieve a true result unbiased by learning effect or repeat performance, volunteers who had received physiotherapy treatment within the last six months or who had any previous experience with, or knowledge of, patella taping were also excluded. Five subjects with unilateral anterior knee pain and five with bilateral anterior knee pain (fifteen knees, six male and four female volunteers with a mean age of 36.93 ±15.04 years) entered and completed the study. A record of their body height and body mass was also obtained. All subjects were screened for any tape allergy a minimum of twenty-four hours prior to testing.

### Measures

Data representing sagittal plane joint angle at the hip, knee and ankle joints of the weight bearing leg during the squat were recorded and analysed within the CODA motion analysis software, which is a specific programme used to analyse bilateral lower-limb movement data. The accuracy of the CODA motion system is well established ( Tyson, 1999 ; Woledge et al., 2005 ), and the lead researcher was practiced in the application and positioning of the specific markers. The maximum joint angle achieved during the squat in each condition was selected within the CODA motion software andfurther analysed within SPSS version 15.0 (Chicago, IL).

Subjective measures of anterior knee pain at rest, and following each single-legged squat under each condition were reported by subjects and recorded on the Numerical Rating Scale (NRS). This outcome measure was used as it has been shown to be the most reliable and valid clinical pain severity tool with excellent sensitivity to change ( Williamson and Hoggart, 2005 ).

### Procedure

Tape was applied to the injured knee in the supine position by the researcher trained in the application of patella tape for AKP. In order to contribute to the internal validity of the study, placebo and patella tape was applied by the same individual throughout the study. A layer of skin protection tape was first applied taking care not to place any tension on the skin. For placebo taping, a single layer “Leukoplast” 3.8cm rigid zinc-oxide tape was applied in line with the under-layer of tape without altering patella alignment or placing the patella under any tension. Patella tape was applied using the same zinc-oxide material from the lateral border of the patella to the medial border with force, thus gliding the patella medially. This protocol was in line with clinical practice and as described in previous literature ( Clark et al., 2000 ; Crossley et al., 2002 ; Ng and Cheng, 2002 ). A second layer was applied to secure the position. For the control condition all tape was removed. Subjects were blinded as to which tape application is used in clinical practice for the management of anterior knee pain, eliminating performance bias in the results.

After being screened for tape allergies, volunteers were required to attend one 30-minute testing session. Subjects were familiarised with the equipment and the test procedure including the single leg squat manoeuvre before data collection commenced. Participants were required to complete a single legged squat, barefoot, on the injured leg with their arms crossed in front of their chest, squatting to as far as they could comfortably before returning to the start position. A continuous movement was required to reflect functional activity. Subjects performed three recorded squats following familiarisation in each condition, one squat with placebo tape, one squat with patella tape and one squat without tape (control). A structured order of testing conditions was implemented so as to minimise interference with the light emitting diodes (LED) markers, which were applied only once for the entire testing procedure, maximising within subject reliability for marker placement. It was hypothesised that by following this order, the placebo tape condition (the middle of the three trials) might produce the greatest depth in squat as a learning effect would have enhanced familiarisation with the expected manoeuvre and fatigue may have influenced the third squat. However, a familiarisation period and adequate timed three minute rest and recovery period followed each squat in order to reduce a learning effect and to avoid muscle fatigue ( Ng and Cheng, 2002 ) and to allow any pain exacerbated to resolve.

**Figure 1:**
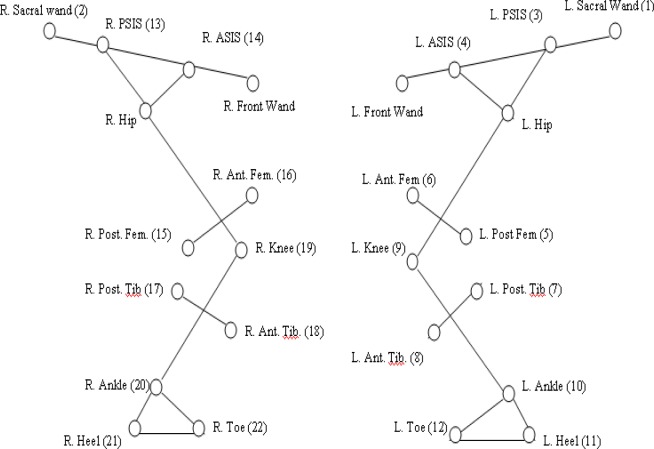
Position of the lower-limb markers for the CODA motion set-up: *Codamotion – Charnwood Dynamics Ltd.
*

Selected kinematic movement data at the hip, knee and ankle joints was recorded using a dual CODA mpx64 (Charnwood Dynamic Ltd., Leicestershire, UK) motion analysis system. CODA mpx64 is a 3-D pre-calibrated system consisting of three optical sensors mounted on a rigid frame within a scanner unit that captures vertical, horizontal and rotational movement. Active LED markers were positioned on each subject as per manufacturer guidelines and in line with previous research ( Maynard et al., 2003 ; Monaghan et al., 2007 ) by the same investigator. Infra-red light signals pulsed sequentially by markers placed on anatomical landmarks of the lower limb, a pelvic frame and thigh and shin wands ( Figure 1 ) were detected by the CODA mpx64 motion analysis system. Kinematic data was acquired and digitised at a sampling rate of 200 Hz for 10 seconds.

### Statistical analysis

Descriptive statistical analysis was performed on the participants’ demographic data.. The Kolmogorov-Smirnov test was carried out on data to assess for normality. Non-parametric statistical analysis was carried out on data found to be not normally distributed. Significant comparisons under each testing condition for changes in pain, maximum joint flexion and squat depth were determined using Friedman Tests (K-Related Samples) and Wilcoxon-Signed Rank Tests. Squat depth was calculated by totaling the maximum degree of flexion at each joint. The level of significance was set at p<0.05.

## Results

### 

#### Demographic Data:

Descriptive statistics for the participants are presented in Table 1 .

The values for maximum flexion at the hip, knee and ankle for each subject during the single-legged squat are displayed in Table 2 . Patella tape produced a statistically significant increase in hip flexion when compared to placebo tape (p=0.008). Statistical significance was found between patella tape and placebo tape (p=0.009), and between no tape and placebo tape (p=0.005) for maximum knee flexion during the squat.

**Figure 2:**
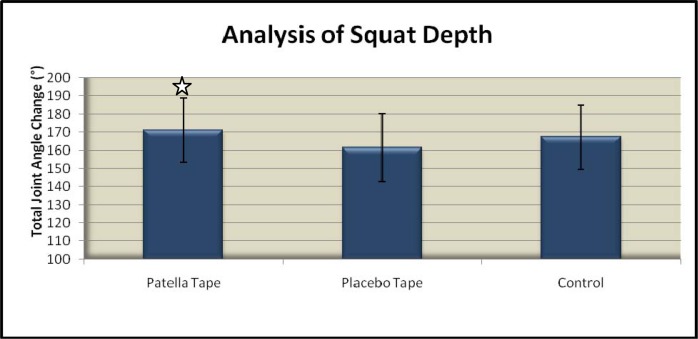
*
Total squat depth (sum of median maximum hip, knee and ankle flexion) under each testing condition* . **
✩** *Indicates statistically significant difference compared to other conditions (p<0.05).
*

Squatting with patella tape resulted in a statistically significant greater overall depth of squat when compared to both placebo tape p=0.008, and no tape p=0.012 ( Figure 2 ).

#### Numerical rating score:

**Figure 3:**
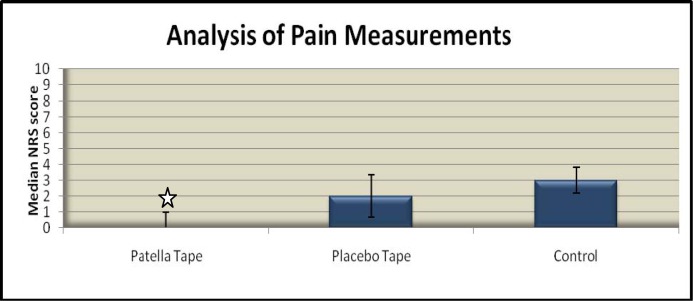
*
Median values on the Numerical Rating Scale for anterior knee pain during the single legged squat under each testing condition* . **
✩** *Indicates statistically significant difference compared to other conditions (p<0.05).
*

Average scores on the Numerical Rating Scale (NRS) for pain during each squat and at rest are reported on in Table 1 and Figure 3 . Data analysis showed a significant reduction in pain when squatting with patella tape compared to placebo (p=0.001) or control conditions (p=0.001). A median NRS of 0 was recorded when squatting with patella tape compared to 2 and 3 with placebo and control respectively. There was no statistically significant difference between squatting with placebo tape compared to squatting with control (p=0.803).

## Discussion

The aim of the current study was to investigate the effects of patella taping on lower-limb sagittal joint kinematics and pain during a single-legged squat. The main findings of this study were that patellar taping produced a significant increase in overall squat depth in the sagittal plane and a decrease in pain compared to both placebo and no tape (control) conditions. Notably, both patella taping and no tape were better than placebo taping in terms of knee joint movement in the sagittal plane. However, only patella taping produced improvements in pain. An improvement in the performance of functional activities following patella taping varies within the current literature ( Gilleard et al., 1998 ; Herrington and Nester, 2004 ; Aminaka and Gribble, 2005 ). Patella taping has been shown to bring about functional gains including improvements during functional closed chain activities ( Somes et al., 1997 ; Gilleard et al., 1998 ; Crossley et al., 2002 ; Cowan et al., 2002 ; Ng and Cheng, 2002 ; Herrington and Nester, 2004 ; Whittingham et al., 2004 ) including squatting activities ( Ng and Cheng, 2002 ) in contrast to no change during a jumping activity ( Ernst et al., 1999 ). Previous research maintains that improved biomechanics at the patellofemoral joint as a consequence of patella taping explains the improvement in function following patellar taping ( Herrington and Nester, 2004 ). It is not known why patellar taping did not increase maximum knee flexion compared to the no tape condition in the current study. A possible hypothesis for this finding is that taping the patellofemoral joint physically restricted the amount of knee flexion achievable at the knee joint compared to the no tape condition, which is supported by the finding that the application of placebo taping produced significantly less maximum knee flexion. A greater number of familiarisation trials may also explain the findings however, this does not explain the reduced range of movement in the placebo trial when familiarisation was optimal. Additionally, taping may have increased proprioceptive input ( Callahan et al., 2002 ), and thus made the subjects more protective of their knee due to the usual knee symptoms associated with this activity. The subjects involved in the study may have been anticipating pain during the task, which caused them to squat to a certain degree of maximum knee flexion during the squat. Pain anticipation is a strategy where the Central Nervous System controls limb movement in people who normally experience chronic pain at that joint even when they are pain-free ( Moseley et al., 2004 ). Indeed, unhelpful pain cognitions are thought to be an important consideration during the interpretation of physical assessments ( Moseley, 2004 ). It was not within the remit of this study to investigate the mechanism by which the patellar taping worked; however, recent studies have established that by taping the patella medially to a more central position within the trochlea groove the point of load application on the patellofemoral joint is changed, improving kinematic control at the knee joint ( Herrington and Nester, 2004 ). Thus, perhaps muscle activation and knee joint control were improved despite no significant increase in knee joint movement in the sagittal plane during the squat compared to the no tape condition.

The results of this current study support previous research, which established that patella tape reduces pain during activities ( Somes et al., 1997 ; Cowan et al., 2002 ; Herrington and Nester, 2004 ; Whittingham et al., 2004 ). Patella taping involved a medial glide patella tape application, which was selected because its use is advocated throughout the literature in the management of AKP ( Somes et al., 1997 ; Clark et al., 2000 ; Crossley et al., 2000 ; 2002 ; Ng and Cheng, 2002 ; Whittingham et al., 2004 ). These findings support the hypothesis that medial glide patella taping produces significant reductions in pain during the single leg squat. McConnell (1986) advocated the theory that by taping the symptomatic patellofemoral joint, the patella is displaced medially to a more central position, re-orienting the bone inside the femoral trochlea groove. The results of this study established that medial glide patella tape produced significant improvements in pain in symptomatic individuals, which may be due to the proposed change in patella alignment that reduces joint stresses and pain ( Crossley et al., 2000 ).

This study did not examine the effects of the patella tape; however, it is proposed that individuals in this study presented with lateral displacement of the patella within the femoral trochlea groove ( Merchant, 1988 ; Pfeiffer et al., 2004 ), and benefited from the patella taping which realigns the patella within the patellofemoral joint in symptomatic patients ( Crossley et al., 2000 ). Patella tape has been proven to produce and maintain a significant medial tilt ( Somes et al., 1997 ; Pfeiffer et al., 2004 ) and medial glide ( Larsen et al., 1995 ) of the patella throughout a brief closed kinetic chain activity resembling the task undertaken in this study. The patellar taping technique employed in this study was synonymous with that described by both Larsen et al. (1995) and Somes et al. (1997) .

The limitations of this study are that the mechanism by which the patella tape affected the musculature around the knee joint and the changes in patella position following patella tape application were not investigated. In addition, an investigation of the duration for the effects of the patellar taping would benefit the clinical application of the results of this study. This information would have further supported the findings and reasoning for the outcomes produced in all three test conditions. Finally, research into pain of people with chronic anterior knee pain may be useful in the interpretation of physical performance and assessment. Further work to examine the kinematic changes in the coronal plane, duration of effect and pain may support the findings of this study, and thus the findings from this study may pave the way for future clinical research in this area.

This study fulfilled the objective of examining the effect of patella tape compared to placebo or no tape on sagittal kinematics and pain in symptomatic knees in a sample of people with AKP. The possible clinical implication of this study is that patella taping may provide a useful adjunct in the rehabilitation of clients with AKP, as it may encourage effective retraining of muscle and movement in a pain free condition. This study also demonstrated that it is important for the clinician to consider the specific movement patterns occurring during the rehabilitation exercises as no significant changes were found between no tape and patellar taping for maximum knee joint flexion during a commonly used exercise i.e. the single leg squat. Despite the results of this study reporting significant findings, the suggested clinical implications need to be considered with caution due to the small sample size of the study. Further larger scale trials using objective imaging would validate the results found in this study and may provide additional information to further inform clinical reasoning when treating people with AKP.

## Conclusion:

Medial glide patella taping contributes to a significantly deeper squat overall on the symptomatic lower limb along with significant reductions in pain compared to placebo tape or no tape condition. Notably, no significant differences were identified for knee range of movement in the patella taping compared to the no tape condition. It is not known why no significant changes in knee joint flexion angles were produced; however, an alteration in patella alignment and subsequent improvements in quadriceps muscle activation are hypothesized to be the likely causative factors for the reduction in pain reported in the patella taped condition.

## Figures and Tables

**Table 1:** *
Demographic characteristics of the participants
*

Gender	6 male, 4 female
Symptomatic knee	8 right, 7 left
Age; years (mean ± SD)	36.93 ± 15.04
Weight; kg (mean ± SD)	81.13 ± 9.92
Height; cm (mean ± SD)	174.87 ± 6.94
NRS at rest (median, range)	1, 0 – 4

**Table 2:** *
Maximum hip, knee and ankle flexion angle (°) achieved under each testing condition* .

Joint/ test condition	Median	Range
Hip Flexion		
Patella Tape	61.12°	44.75° – 82.14°
Placebo Tape	55.09°	45.32° – 78.25°
Control	54.9°	48.14° – 75.29°
Knee Flexion		
Patella Tape	77.55°	62.47° – 90.23°
Placebo Tape	69.43°	60.42° – 85.87°
Control	80°	60.63° – 87.49°
Ankle Dorsiflexion		
Patella Tape	31.77°	20.15 – 40.69°
Placebo Tape	31.44°	19.35° – 38.13°
Control	32.4°	17.82° – 39.57°
